# A Novel Binary Dream Optimization Algorithm with Data-Driven Repair for the Set Covering Problem

**DOI:** 10.3390/biomimetics11030197

**Published:** 2026-03-09

**Authors:** Broderick Crawford, Hugo Caballero, Gino Astorga, Felipe Cisternas-Caneo, Marcelo Becerra-Rozas, Alan Baeza, Gabriel Bernales, Pablo Puga, Giovanni Giachetti, Ricardo Soto

**Affiliations:** 1 Escuela de Ingeniería Informática, Pontificia Universidad Católica de Valparaíso, Avenida Brasil 2241, Valparaíso 2362807, Chilealan.baeza.o@mail.pucv.cl (A.B.); gabriel.bernales.a@mail.pucv.cl (G.B.);; 2 Escuela de Negocios Internacionales, Universidad de Valparaíso, Alcalde Prieto Nieto 452, Viña del Mar 2572048, Chile; 3 Facultad de Ingeniería, Universidad Andres Bello, Antonio Varas 880, Providencia, Santiago 7591538, Chile

**Keywords:** set covering problem, metaheuristics, dream optimization algorithm, repair strategies, machine learning–based control, combinatorial optimization, 68T20, 68W25, 90C27, 90C59, 68Q25

## Abstract

The Set Covering Problem is a fundamental NP-hard problem in combinatorial optimization and plays a central role in a wide range of industrial decision-making processes, including logistics planning, scheduling, facility location, network design, and resource allocation. In many real-world contexts, problems of this type are large in scale and highly constrained, which makes exact solution methods computationally impractical and encourages the use of metaheuristic approaches capable of producing high-quality solutions within limited time budgets. In this work, we propose a discrete adaptation of the Dream Optimization Algorithm, focusing on the challenges that emerge when algorithms originally designed for continuous search spaces are applied to binary and strongly constrained models. The continuous search process is mapped onto the binary decision space through a fixed discretization scheme. As a consequence of this transformation, some constraints may not be met, underscoring the importance of effective feasibility restoration mechanisms. Because the discretization stage may produce infeasible solutions and frequently induces plateaus that hinder further improvement, an explicit repair phase becomes necessary to restore feasibility and promote effective search progression. To strengthen this process, the study introduces an adaptive control mechanism based on bandit driven operator selection, which dynamically chooses among different repair procedures during the search. Experimental results on benchmark instances show that the proposed approach consistently achieves high quality solutions with low relative deviation from known optima and stable behavior across independent runs.

## 1. Introduction

Metaheuristic algorithms have become a widely adopted alternative for tackling complex optimization problems that arise in real world applications, particularly in situations where classical mathematical programming approaches fail to provide solutions within acceptable computational times [1]. These problems are often characterized by large decision spaces, multiple constraints, and strong combinatorial complexity, which rapidly renders exact methods impractical even for instances of moderate size [2]. Such difficulties are especially apparent in industrial contexts including logistics, telecommunications, and manufacturing, where the growing presence of Internet of Things technologies increases the demand for optimization strategies that are both robust and computationally efficient [3,4].

In this work, a version of the Dream Optimization Algorithm (DOA) adapted to solve the Set Covering Problem (SCP) is presented. This algorithm is inspired by the rapid eye movement (REM) sleep phase, in which solutions are fragmented and reorganized to generate new combinations, allowing large exploratory jumps and reducing the risk of premature convergence to local optima [5]. DOA was selected primarily because its original study reports competitive or superior performance on standard CEC benchmark suites with a reduced number of control parameters, making it a robust and well-validated baseline for methodological extensions.

To enable DOA to operate on the SCP, two essential components are required: a binarization mechanism and a solution repair phase. While binarization allows exploration of the binary search space, it may generate infeasible solutions that violate coverage constraints  [6,7]. Therefore, a repair mechanism becomes necessary to restore feasibility. The effectiveness of repair operators depends strongly on the structural characteristics of each instance, and relying on a single fixed strategy may limit the performance and robustness of the optimization process.

To address this limitation, the repair phase is treated as an adaptive component of the search process. A multi-armed bandit (MAB) mechanism [8] is employed to dynamically select the most appropriate repair operator from a predefined set according to its observed contribution to solution quality. This strategy is consistent with principles widely explored in adaptive operator selection and hyperheuristic frameworks. From a biomimetic perspective, this adaptive selection mechanism reflects reinforcement dynamics observed in biological systems, where behavioral strategies that lead to improved outcomes tend to be strengthened over time, while less effective responses are progressively suppressed. In this way, the learning component operates at the decision level, guiding the selection of repair operators without altering the discretization scheme or the core dynamics of the algorithm.

In this context, the present work makes three main contributions. First, it proposes a binary adaptation of the Dream Optimization Algorithm for the Set Covering Problem, addressing the challenges that arise when a metaheuristic originally designed for continuous search spaces is applied to a binary and highly constrained combinatorial problem. Second, the study introduces an adaptive feasibility restoration mechanism in which the repair phase is treated as an active component of the search process and is dynamically managed through a multi armed bandit strategy, allowing different repair operators to be selected according to their observed contribution to the optimization. Third, the proposed approach is assessed through an extensive experimental evaluation on standard SCP benchmark instances, enabling a detailed analysis of solution quality, robustness, and the role played by adaptive repair control in the overall performance of the algorithm.

The remainder of this paper is organized as follows. Section 2 introduces the SCP and its main industrial applications. Section 3 describes the feasibility restoration phase. Section 4 presents the original DOA and its exploration and exploitation mechanisms. Section 5, defines the binarization scheme adopted in this study. Section 6 details the proposed binary adaptation of DOA, including the validation and repair mechanism. Section 7 presents the experimental results and a descriptive comparison with several well established metaheuristic methods. Section 8 summarizes the main novelties of the proposed approach for solving the SCP. Finally, Section 9 presents the main conclusions and Section 10 discusses possible extensions of the proposed methodology.

## 2. Set Covering Problem

The Set Covering Problem is a classical combinatorial optimization problem that belongs to the class of NP-hard problems [9]. It involves selecting a minimal subset of sets, from a given collection, whose union covers all elements of a given universe. The ultimate goal is to ensure that no element of this universe remains uncovered while minimizing the overall selection cost. This characteristic makes the problem particularly relevant for both theoretical optimization research and applications in real world environments.

In the last two years, research on the Set Covering Problem has continued to emphasize binary adaptations of population-based metaheuristics, with particular attention to binarization mechanisms such as transfer functions and discretization rules, and their impact on solution quality [10,11]. Recent studies report binary SCP solvers that integrate transfer functions, elitist discretization schemes and diversification mechanisms to enhance exploration capabilities and robustness across benchmark instances [12,13]. Complementarily, other works have conducted systematic experimental analyzes of binarization and transfer function choices within binary metaheuristics for the SCP, consistently highlighting discretization design as a critical determinant of algorithmic performance [6].

### 2.1. Mathematical Formulation

Formally, the SCP can be defined mathematically as follows; Let U={1,2,…,n} be a universal set consisting of *n* elements. We also have a collection of *m* subsets $S=\{S_{1},S_{2},\ldots ,S_{m}\}$, where each subset $S_{j}\subseteq U$ contains some elements of the universal set and has an associated cost $c_{j}$. With this, we must find a subcollection of subsets from *S* such that every element in *U* is covered by at least one selected subset while also minimizing the cost.

This is done by introducing binary decision variables $x_{j}$, which indicate whether a subset $S_{j}$ is selected or not. Their purpose is to allow the model to identify which subsets must be included to cover all elements of the set *U*. These variables are defined as follows:

$$x_{j}=\left\{\begin{array}{cc} 1, & \mathrm{if}\;\mathrm{the}\;\mathrm{subset}\;S_{j}\;\mathrm{is}\;\mathrm{selected},\\ 0, & \mathrm{otherwise}. \end{array}\right.$$

Based on the elements defined above, the SCP can be computationally represented as a binary matrix $A=\left[a_{ij}\right]\in {\left\{0,1\right\}}^{n\times m}$, where each row corresponds to an element in *U* and each column corresponds to a subset in *S*, and the entries indicate whether an element belongs to a subset, as follows:

$$A=\left[\begin{array}{cccc} a_{1,1} & a_{1,2} & \ldots & a_{1,m}\\ a_{2,1} & a_{2,2} & \ldots & a_{2,m}\\ \vdots & \vdots & \ddots & \vdots \\ a_{n,1} & a_{n,2} & \ldots & a_{n,m} \end{array}\right]$$

The SCP is then formulated as the following integer linear program. The objective function in Equation (1) minimizes the total cost associated with the selected subsets, as indicated by the binary decision variables $x_{j}$. Constraint Equation (2) ensures that each element *i* is covered by at least one selected subset, while constraint Equation (3) enforces the binary nature of the decision variables.

(1) $$\mathrm{Minimize}\;Z=\sum_{j=1}^{m}c_{j}x_{j}$$

(2) $$\sum_{j=1}^{m}a_{i,j}x_{j}\geq 1,\;\forall i=1,\ldots ,n$$

(3) $$x_{j}\in \left\{0,1\right\},\;\forall j=1,\ldots ,m$$

### 2.2. Practical Application in Business

Optimization is of great importance in modern industry, as it is in decision-making and the proper use of resources [14] as well as in logistics and network design in general [15,16,17,18,19].

A practical example of the use of the SCP appears in marketing, an area that has experienced strong growth in recent years due to the diversity of communication channels and the limited availability of resources. Companies seek to maximize the efficiency of their investments; therefore, they must decide which channels to use—such as social networks, television, radio, among others—to reach target customer segments at the lowest possible cost. To formulate this problem, we define the set of market segments to be covered in Table 1, and the set of media channels available to reach a specific audience at minimum cost, as shown below:

### 2.3. Illustrative SCP Formulation for a Marketing Application

Let

(4) E={Youth,Adults,Professionals,Families,Elderly},

be the set of customer segments to be covered, and

(5) S={TV,Radio,Magazine,Instagram,Facebook,Daily},

the set of available advertising media.

Each medium j∈S covers a subset $C_{j}\subseteq E$, defined as

(6) $$\begin{array}{cc} C_{\mathrm{TV}} & =\{\mathrm{Youth},\;\mathrm{Adults},\;\mathrm{Families}\}, \end{array}$$

(7) $$\begin{array}{cc} C_{\mathrm{Radio}} & =\{\mathrm{Adults},\;\mathrm{Elderly}\}, \end{array}$$

(8) $$\begin{array}{cc} C_{\mathrm{Magazine}} & =\{\mathrm{Professionals},\;\mathrm{Adults}\}, \end{array}$$

(9) $$\begin{array}{cc} C_{\mathrm{Instagram}} & =\{\mathrm{Youth},\;\mathrm{Professionals}\}, \end{array}$$

(10) $$\begin{array}{cc} C_{\mathrm{Facebook}} & =\{\mathrm{Youth},\;\mathrm{Adults},\;\mathrm{Families},\;\mathrm{Elderly}\}, \end{array}$$

(11) $$\begin{array}{cc} C_{\mathrm{Daily}} & =\{\mathrm{Adults},\;\mathrm{Families}\}. \end{array}$$

A binary decision variable is associated with each medium:

(12) $$x_{j}=\left\{\begin{array}{cc} 1, & \mathrm{if}\;\mathrm{medium}\;j\;\mathrm{is}\;\mathrm{selected},\\ 0, & \mathrm{otherwise}, \end{array}\right.\;\forall j\in S.$$

The objective is to minimize the total advertising cost:

(13) $$\min Z=100\;x_{\mathrm{TV}}+40\;x_{\mathrm{Radio}}+60\;x_{\mathrm{Magazine}}+50\;x_{\mathrm{Instagram}}+70\;x_{\mathrm{Facebook}}+30\;x_{\mathrm{Daily}}.$$

Subject to the coverage constraints:

(14) $$\begin{array}{cccc} x_{\mathrm{TV}}+x_{\mathrm{Instagram}}+x_{\mathrm{Facebook}} & \geq 1, & \; & \left(\mathrm{Youth}\right) \end{array}$$

(15) $$\begin{array}{cccc} x_{\mathrm{TV}}+x_{\mathrm{Radio}}+x_{\mathrm{Magazine}}+x_{\mathrm{Facebook}}+x_{\mathrm{Daily}} & \geq 1, & \; & \left(\mathrm{Adults}\right) \end{array}$$

(16) $$\begin{array}{cccc} x_{\mathrm{Magazine}}+x_{\mathrm{Instagram}} & \geq 1, & \; & \left(\mathrm{Professionals}\right) \end{array}$$

(17) $$\begin{array}{cccc} x_{\mathrm{TV}}+x_{\mathrm{Facebook}}+x_{\mathrm{Daily}} & \geq 1, & \; & \left(\mathrm{Families}\right) \end{array}$$

(18) $$\begin{array}{cccc} x_{\mathrm{Radio}}+x_{\mathrm{Facebook}} & \geq 1, & \; & \left(\mathrm{Elderly}\right) \end{array}$$

(19) $$x_{j}\in \left\{0,1\right\},\;\forall j\in S.$$

The result indicates that the marketing department does not need to invest in all available channels; instead, only those that cover the required customer segments should be selected. This approach optimizes the allocated budget and achieves maximum coverage at minimum cost. This practical example can be extended to advertising media selection, promotional strategies at different times, customer sampling for surveys, or campaign coverage assessments. The example illustrates any problem that aims to meet minimum while minimizing the cost of available resources.

## 3. Repair Phase

The repair phase has received limited attention in the literature, as it is commonly assumed to be implemented through a single, fixed method. Nevertheless, it has a significant impact on solution quality in the SCP, since it determines how infeasible solutions are transformed into feasible ones, directly influencing both the objective function value and the regions of the feasible space explored by the algorithm [20,21]. Previous studies on SCP and constrained metaheuristics have shown that repair strategies are not neutral operations, as they modify the structure and cost of solutions while conditioning the search dynamics [22,23,24]. Consequently, the design and activation of repair mechanisms play a critical role in preserving solution quality, avoiding systematic cost overruns and mitigating stagnation during the search process.

### 3.1. Role of the Repair Phase in the SCP

In the SCP, let U={1,2,…,n} denote the universe set of elements and $S=\{S_{1},S_{2},\ldots ,S_{m}\}$ the collection of available subsets. A solution is valid only if all elements in *U* are covered by at least one selected subset. However, when discretized metaheuristic approaches are employed, it is common for some generated solutions to violate this condition. The repair process is therefore designed to correct such situations by adding subsets to the solution until full coverage is restored [9]. From a mathematical perspective, a solution is infeasible if there exists at least one element i∈U such that meaning that no selected subset covers that element.

(20) $$\sum_{j=1}^{m}a_{ij}x_{j}=0,$$

The repair mechanism operates by identifying these uncovered elements and selecting additional subsets that include them, updating the decision variables $x_{j}$ until the coverage constraint is satisfied for all elements [25,26,27,28].

### 3.2. Repair Strategies Adopted in This Study

The repair mechanisms employed in this study follow classical greedy heuristics for the SCP, particularly those described by Lan et al. [29], where feasibility is restored by iteratively adding subsets according to cost and coverage criteria until all coverage constraints are satisfied. These operators can be mapped in the general categories and shows in Table 2. This taxonomy is supported by foundational studies in metaheuristic design and hybridization frameworks, which emphasize the role of operator behavior in steering search dynamics [30,31].

The repair operator set was designed following established principles in adaptive operator selection and hyper-heuristic frameworks, where the objective is not to identify a single dominant operator, but to enable adaptive control over operators exhibiting distinct structural behaviors [27,33]. By including deterministic, penalized, stochastic, and hybrid repair strategies, the proposed set captures complementary feasibility restoration patterns commonly reported in SCP and constrained metaheuristics. The Table 3 shows the repair operators used in this study.

Section 3.3 presents an Adaptive Operator Selection strategy to control this phase of the algorithm.

### 3.3. Adaptive Operator Selection Strategy

Adaptive Operator Selection (AOS) is a decision mechanism implemented through a multi-armed bandit learning model, designed to dynamically choose among a predefined set of operators during the execution of a metaheuristic. Rather than relying on a fixed strategy, AOS updates the expected utility of each operator based on its observed contribution to the search process. In the proposed approach, AOS is employed to control the repair phase, selecting at each iteration the operator to be applied whenever an infeasible solution is generated.

The bandit controller operates in two complementary phases: a selection phase, in which a repair operator is chosen, and an update phase, in which its contribution to the search process is learned. At iteration *t*, a repair operator $a_{t}$ is selected according to an ε-greedy policy. Let u∼U(0,1). The repair operator is selected as

(21) $$a_{t}=\left\{\begin{array}{cc} \mathrm{random}\;\mathrm{operator}\;\mathrm{from}\;R, & \mathrm{if}\;u<\varepsilon ,\\ \arg\max_{a\in R}Q\left(a\right), & \mathrm{otherwise}. \end{array}\right.$$

The instantaneous reward associated with the selected repair operator is defined as the absolute improvement in the global best solution obtained after its application. Let $f_{\mathrm{best}}^{\left(t\right)}$ denote the best objective value found up to iteration *t*. The reward is computed as

(22) $$r_{t}=f_{\mathrm{best}}^{\left(t-1\right)}-f_{\mathrm{best}}^{\left(t\right)}.$$

This formulation is conceptually aligned with the fitness improvement-based credit assignment strategy described by Fialho et al. [33]. The estimated value of the selected operator is then updated using the standard sample-average incremental rule widely adopted in multi-armed bandit and reinforcement learning literature [35]:

(23) $$Q\left(a_{t}\right)\leftarrow Q\left(a_{t}\right)+\frac{r_{t}-Q\left(a_{t}\right)}{N(a_{t})}.$$

Together, Equations (21)–(23) define a bandit-based adaptive operator selection mechanism that learns which repair strategies contribute most effectively to global optimization progress. The Table 4 explains the notation used in these equations.

Algorithm 1 defines the AOS mechanism used in this study. The method is implemented as a multi-armed bandit with an ε-greedy policy. At each iteration, one operator is selected, applied within the main algorithm, and its expected reward is updated based on the observed outcome.
**Algorithm 1** Generic Adaptive Operator Selection (multi-armed bandit).**Require:** Operator set $O=\{o_{1},\ldots ,o_{K}\}$, exploration rate ε
1:Initialize $Q(o_{k})\leftarrow 0$, $N(o_{k})\leftarrow 0$ for all $o_{k}\in O$2:**for** t=1 to *T* **do**3:   Draw u∼U(0,1)4:   **if** u<ε **then**                ▹ Selection phase5:   Select $o_{t}$ uniformly at random from O6:   **else**7:   Select $o_{t}=\arg\max_{o_{k}\in O}Q\left(o_{k}\right)$8:   **end if**9:   Execution phase, defined by the underlying optimization problem10: Apply operator $o_{t}$ within the main algorithm11: Compute reward $r_{t}$ using Equations (21) and (23) ▹ Update phase12:**end for**

## 4. Dream Optimization Algorithm

The Dream Optimization Algorithm is a bio-inspired metaheuristic developed by Yifan Lang and Yuelin Gao [5] to find optimal solutions in continuous search spaces. The method draws its inspiration from the neurological processes that occur during sleep, particularly during the stage of rapid eye movement (REM), in which memory retention, partial forgetting, and self organization of experiences emerge as dreams. Based on this biological analogy, DOA integrates three complementary strategies that mimic these cognitive phenomena throughout its search process: the memory strategy, the forgetting and supplementation strategy, and the dream sharing strategy. Together, they coordinate the balance of the algorithm between global exploration and local exploitation.

The Dream Optimization Algorithm was selected because its original study reports competitive or superior performance on standard CEC benchmark suites (CEC2017, CEC2019, and CEC2022) with a reduced number of control parameters. These results, obtained from rigorous comparisons against 27 classical, recent, and high-performing metaheuristic algorithms (see Section 4.3 and Table 2 in  [5]), support the use of DOA as a well-validated baseline for methodological extensions.

A key element of the algorithm is the transition factor α, which determines how the total number of iterations $T_{\max}$ is divided between the exploration and exploitation phases. Specifically, the number of exploration iterations $T_{d}$ is calculated as follows:

(24) $$T_{d}=\alpha \times T_{\max}$$

This formulation allows the algorithm to precisely adjust the length of each phase, ensuring a controlled transition from global search to local refinement as the optimization progresses.

### 4.1. Initialization Phase

DOA is a population-based metaheuristic, the optimization process begins by defining an initial population of candidate solutions. Each individual represents a point in the continuous search space and is randomly generated within the predefined lower and upper bounds of the problem domain.

(25) $$X_{i}=X_{l}+\mathrm{rand}\times \left(X_{u}-X_{l}\right),\;i=1,2,\ldots ,N,$$

where *N* represents the number of individuals, i.e., the population size; $X_{i}$ is the *i*th individual in the population; $X_{l}$ and $X_{u}$ represent the lower and upper boundaries of the search space, respectively; rand is a Dim dimensional vector, with each dimension being a random number between 0 and 1; the obtained population can be represented as follows:

(26) $$X=\left[\begin{array}{c} X_{1}\\ X_{2}\\ \vdots \\ X_{i}\\ \vdots \\ X_{N} \end{array}\right]=\left[\begin{array}{cccc} x_{1,1} & x_{1,2} & \ldots & x_{1,Dim}\\ x_{2,1} & x_{2,2} & \ldots & x_{2,Dim}\\ \vdots & \vdots & \ddots & \vdots \\ x_{i,1} & x_{i,2} & \ldots & x_{i,Dim}\\ \vdots & \vdots & \ddots & \vdots \\ x_{N,1} & x_{N,2} & \ldots & x_{N,Dim} \end{array}\right],$$

where $x_{i,j}$ represents the position of the *i*th individual in the *j*th dimension, and Dim represents the dimensionality of the optimization problem.

### 4.2. Exploration Phase

The exploration phase represents the stage in which the algorithm expands its search to new regions of the solution space, without yet focusing on the most promising results. This phase emulates the cognitive process of human dreaming, where the brain combines memories and experiences to generate new associations; in this stage, the individuals in the population update their positions through a combination of the best known solutions and random components, which promotes diversity and prevents premature convergence within a single region of the search space. The forgetting and supplementation strategy predominates in this process, introducing controlled variations that help the algorithm escape local optima and discover unexplored areas. Compared with conventional metaheuristics, DOA exhibits a more stable balance between exploration and exploitation, a lower dependency on control parameters, and a smoother search dynamics. Moreover, its design—rooted in cognitive processes rather than physical or biological analogies—provides greater flexibility to adapt to complex and multi objective optimization problems, achieving faster convergence and higher solution accuracy.

#### 4.2.1. Memory Based Update Strategy

This strategy is based on keeping and reusing useful information obtained during the optimization process. Before the individuals in a group enter the “dreaming” phase, the position of the best individual $X_{{\mathrm{best}}_{q}}^{t}$ found by that group is taken as a reference. Then, the positions of all individuals $X_{i}^{t+1}$ are updated with the coordinates of this best solution. In this way, each member starts the next iteration from a promising region of the search space. This approach allows the algorithm to take advantage of high quality information discovered in earlier stages and to strengthen the exploitation phase by guiding the population toward better areas. This is shown in Equation (27).

(27) $$X_{i}^{t+1}=X_{\mathrm{bestq}}^{t}$$

*X* represents the position of an individual within the search space. The term *i* identifies each individual in the population, allowing the algorithm to distinguish among the different solutions evaluated in each cycle. Finally, *t* denotes the iteration number.

#### 4.2.2. Forgetting and Supplementation Strategy

This technique introduces variability into the search by allowing individuals $x_{ij}^{t+1}$ to forget certain dimensions *j* of their position, which are then replaced using information from the best individual of the group $x_{\mathrm{bestj}}^{t}$ along with a random component that recalculates the final value composed of the lower $x_{lj}$ and upper $x_{uj}$ bounds of the problem and adjusts its value depending on the current iteration *t*, promoting both global exploration and local exploitation. This is modeled by Equation (28).

(28) $$x_{i,j}^{t+1}=x_{\mathrm{best}q,j}^{t}+\left(x_{l,j}+\mathrm{rand}\times \left(x_{u,j}-x_{l,j}\right)\right)\times \frac{1}{2}\times \left(\cos\;\left(\pi \times \frac{t+T_{\max}-T_{d}}{T_{\max}}\right)+1\right)$$

where

$T_{\max}$: total number of iterations in the optimization process.
$T_{d}$: number of iterations assigned to the exploration phase.
rand: random value uniformly distributed in the range [0, 1], introducing variability and promoting search diversity.
$\cos\left(\pi \times \frac{t+T_{\max}-T_{d}}{T_{\max}}\right)$: cosine function that regulates the transition between exploration and exploitation, gradually reducing the effect of randomness as the algorithm progresses.
*i*: index identifying each individual or agent within the population.
*j*: index representing a specific dimension of the search space.
$k_{q}$: set of dimensions to be forgotten and replaced in each group *q*, maintaining diversity and preventing premature convergence.

Furthermore, forgetting dimensions $k_{q}$ are unique for each group *q*, and fluctuate depending on both the dimensions of the problem Dim and a random component randi that delimits the dimensions to forget between a range, encouraging diversity and preventing premature convergence. This is shown in Equation (29).

(29) $$k_{q}=\mathrm{randi}\left(\left\lfloor \frac{\mathrm{Dim}}{8\times q}\right\rfloor ,\max\left\{2,\left\lfloor \frac{\mathrm{Dim}}{3\times q}\right\rfloor \right\}\right),\;q=1,2,3,4,5$$

#### 4.2.3. Dream Sharing Strategy

This technique is based on the random exchange of information between individuals $x_{ij}^{t+1}$, allowing them to acquire knowledge from other members $x_{mj}$ by utilizing their position *m* within the population $N_{max}$ in certain selected forgetting dimensions *j*, depending on the group *q* to which the individual belongs. Notably, the forgetting dimensions are calculated in the same manner as in the Forgetting and Supplementation strategy, already shown in Equation (29). Ultimately by sharing parts of their “dreams”, the algorithm avoids becoming prematurely trapped in local optima and promotes a broader and more efficient exploration of the solution space. This can be seen below in Equation (30)

(30) $$x_{ij}^{t+1}=\left\{\begin{array}{cc} x_{mj}^{t+1}, & m\leq i\\ x_{mj}^{t}, & i<m\leq N_{max} \end{array}\right.,\;j=K_{1},K_{2},\ldots ,K_{k_{q}}$$

### 4.3. Exploitation Phase

The exploration phase of the DOA begins by dividing the population into five groups, each characterized by distinct memory capacities. During this stage, every iteration is treated as a dreaming process, where individuals update their positions by referencing the best solution previously obtained by their own group. Similar to human dreaming, part of the stored information is intentionally forgotten and replaced with new elements, allowing the algorithm to explore unvisited regions of the search space. The number of forgotten dimensions varies among groups and is determined by the parameters $k_{1},k_{2},k_{3},k_{4},k_{5}$, which represent different levels of memory retention. Specifically, each individual first resets its position to that of the best member within its group, and then $k_{q}$ dimensions are randomly selected from the total problem dimensions (Dim) and updated. This mechanism encourages population diversity, prevents premature convergence, and enables the algorithm to maintain a dynamic balance between creativity and memory during the exploration process.

#### 4.3.1. Memory Strategy

This technique once again utilizes information from the best individual previously obtained $X_{\mathrm{best}}$. The crucial difference now is that it is applied to the entire population $X_{i}^{t+1}$ before the “Dreaming” process. This naturally favors convergence, guiding the entire population toward the best known solution. This can be seen below in Equation (31)

(31) $$X_{i}^{t+1}=X_{\mathrm{best}}^{t}$$

#### 4.3.2. Forgetting and Supplementation Strategy

Similar to its use in the exploration phase, this technique is responsible for introducing perturbations in the forgetting dimension *j*. However, in the exploitation phase, these perturbations are applied to the entire population $x_{ij}^{t+1}$. The best individual $x_{\mathrm{best},j}^{t}$ acts as a base to replace the forgotten values, maintaining a delicate balance between intensive exploitation of promising areas and preventing the total loss of diversity within the population, helping avoid premature convergence to local optima, as illustrated in Equation (32).

(32) $$x_{ij}^{t+1}=x_{\mathrm{best},j}^{t}+(x_{ij}+\mathrm{rand}\times \left(x_{uj}-x_{lj}\right))\times \frac{1}{2}\times \left(\cos\left(\pi \times \frac{t}{T_{\max}}\right)+1\right)$$

where

$x_{ij}^{t+1}$: updated position of individual *i* in dimension *j* at iteration t+1, representing the new solution after the exploitation step.
$x_{\mathrm{best},j}^{t}$: best position found so far in dimension *j* at iteration *t*, used as a reference to guide convergence toward promising regions.
$x_{ij}$: current position of individual *i* in dimension *j* before applying the update rule.
$x_{uj}$: position of another individual *u* (different from *i*) in dimension *j*, randomly selected from the same group to introduce additional variation.
$T_{\max}$: maximum number of iterations, used to normalize the cosine-based modulation term.
*i*: index representing the individual or agent within the population.
*j*: index identifying a specific dimension of the search space.
*u*: index of another individual selected randomly from the same group, used to create the differential perturbation $\left(x_{uj}-x_{ij}\right)$.

Moreover, forgetting dimensions $k_{r}$ serve the same goal as in the exploitation phase, and are calculated similarly using the problem dimension Dim within a defined randi range. However, in the absence of groups, these dimensions are applied uniformly across the entire population, as shown in Equation (33).

(33) $$k_{r}=\mathrm{randi}\left(2,\max\left\{2,\left\lfloor \frac{\mathrm{Dim}}{3}\right\rfloor \right\}\right)$$

### 4.4. Algorithm Pseudo Code

The Algorithm 2 presents the pseudocode of DOA, outlining the overall process, the required input parameters, and the resulting outputs.
**Algorithm 2** Dream Optimization Algorithm (DOA) with explicit exploration and exploitation phases.**Input:** Population size $N_{\max}$, lower bounds $X_{l}$, upper bounds $X_{u}$, problem dimension Dim, current iteration *t*, demarcation $T_{d}$, maximum iterations $T_{\max}$, forgetting dimensions $\left(k_{1},\ldots ,k_{q}\right)$.**Output:** Best solution $X_{\mathrm{best}}$ and minimum fitness $Fitness_{\mathrm{best}}$.
1: Generate initial population *X* using Equations (24) and (25)2: Check solution bounds3: Evaluate fitness4: Determine $X_{\mathrm{best}}$ and $Fitness_{\mathrm{best}}$5: t←16: **while** $t<T_{d}$ **do**                   ▹ Main loop7:    Update $X_{\mathrm{best}}$ and $Fitness_{\mathrm{best}}$8:    **for** q=1:5 **do**9:       Update $X_{\mathrm{best}q}$ and $Fitness_{\mathrm{best}q}$10:      Update $k_{q}$ using Equation (29)11:      Update $X^{t+1}$ using Equation (27)12:      $\left(K_{1},\ldots ,K_{k_{q}}\right)\leftarrow randperm\left(k_{q},N\right)$13:      **for** $i=\left(\left(q-1\right)\frac{N}{5}+1\right):\left(q\frac{N}{5}\right)$ **do**14:         **if** rand<u **then**15:            **Exploration:** update $x_{i,j}$ using Equation (28)16:            Check bounds17:         **else**18:            **Exploitation:** update $x_{i,j}$ using Equation (32)19:         **end if**20:      **end for**21:   **end for**22:   t←t+123:**end while**24:**while** $t\geq T_{d}$ **and** $t<T_{\max}$ **do**25:   Update $k_{r}$ using Equation (33)26:   Update $X^{t+1}$ using Equation (27)27:   $\left(K_{1},\ldots ,K_{k_{r}}\right)\leftarrow randperm\left(k_{r},N\right)$28:   **for** i=1:N **do**29:      **Exploitation:** update $x_{i,j}$ using Equation (32)30:      Check bounds31:   **end for**32:   t←t+133:**end while**

## 5. Binarization Scheme

A preliminary parameter setting phase was conducted in order to identify the most appropriate discretization scheme for adapting DOA to the binary search space of the SCP. Several combinations of transfer functions and binarization rules were evaluated under identical computational conditions. The experimental results [36] consistently indicated that the V3-ELIT discretization scheme achieved the best trade off between solution quality and search stability across the tested instances.

An additional reason for selecting V3-ELIT is its more consistent probabilistic behavior under frequent repair operations, which are typical in highly constrained problems such as the SCP. Previous studies have shown that S-type transfer functions tend to rapidly saturate probabilities toward 0 or 1, leading to premature loss of diversity and stagnation, particularly when combined with repeated feasibility restoration mechanisms [12,37]. In contrast, V-type transfer functions exhibit smoother probability transitions, which interact more stably with elitist discretization and repair mechanisms, reducing disruptive effects between exploration, exploitation, and feasibility restoration. This behavior has been empirically observed in comparative binarization studies, where V-type functions combined with elitist rules consistently show lower variability and more controlled search dynamics under constraint-handling scenarios [36,37].

**Figure 1:**
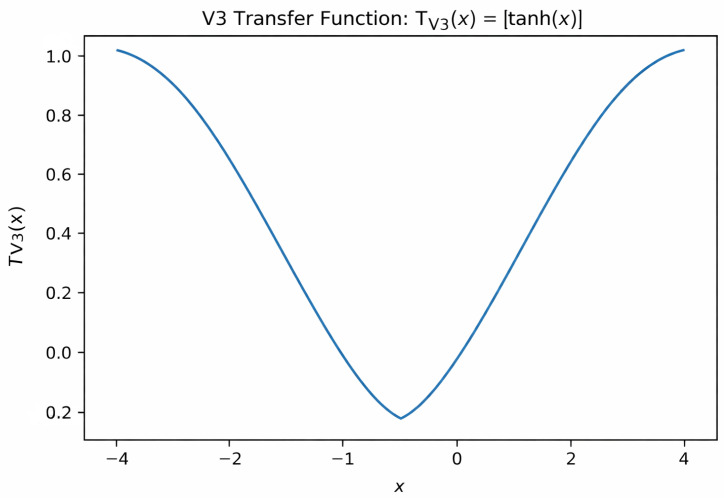
V3 transfer function $T_{V3}\left(x\right)=\left|\tanh\left(x\right)\right|$, used to map continuous values to [0,1]. Larger absolute values increase the probability of changing a binary variable, while values near zero preserve the current state.

The V3 transfer function (Figure 1) belongs to the family of V–shaped transfer functions and is defined as

$$T_{V3}\left(x\right)=\left|\tanh(x)\right|$$

This function maps continuous values into the interval (0,1), where larger absolute values indicate higher probabilities of bit flipping, thus promoting balanced exploration in the binary space.

The ELIT binarization rule updates each binary decision variable by comparing the output of the transfer function with the corresponding bit of the current global best solution. Specifically, a binary variable $b_{i,j}$ is updated as

$$b_{i,j}=\left\{\begin{array}{cc} b_{j}^{\mathrm{best}}, & \mathrm{if}\;\mathrm{rand}<T_{V3}\left(x_{i,j}\right),\\ 1-b_{j}^{\mathrm{best}}, & \mathrm{otherwise}. \end{array}\right.$$

This elitist mechanism biases the discretization process toward the structure of the best solution found so far, improving convergence while preserving diversity through stochastic perturbations.

Based on this evidence, V3-ELIT was fixed for all subsequent experiments, allowing the effect of the adaptive repair mechanism to be isolated and ensuring a fair and controlled evaluation of the proposed learning–based repair selection framework.

## 6. Binary DOA

In this section, we present the Binary DOA algorithm, which addresses the binary nature of the problem and enables the resolution of the SCP. This version integrates three main components. The core DOA (Section 4), which operates in a continuous space and provides the exploration and exploitation dynamics. Second, the binarization process (Section 5), which is applied to the proposed solution, and finally, since binarization may generate infeasible solutions, a feasibility restoration (Section 3) phase is incorporated, in which a set of repair operators is adaptively managed through a multi-armed bandit (MAB) mechanism. The bandit acts as a high-level decision controller, selecting the most appropriate repair operator according to its observed contribution to the overall search progress at each iteration.

### 6.1. Core DOA

The core of the DOA is defined by the continuous position update mechanism that generates new candidate solutions through the alternating application of exploration and exploitation operators. This core is responsible for transforming the current population into a new candidate population at each iteration and constitutes the main search engine of the algorithm. Algorithm 3 describes the core of the DOA.
**Algorithm 3** Core generation mechanism of DOA.**Input:** Current population $X^{t}$, iteration *t*, demarcation iteration $T_{d}$, maximum number of iterations $T_{\max}$, forgetting parameters $\left(k_{1},k_{2},k_{3},k_{4},k_{5},k_{r}\right)$, and controlparameter *u*.**Output:** New candidate continuous population $X^{t+1}$.
1: **if** $t<T_{d}$ **then**           ▹ Exploration–exploitation stage2:    **for** q=1:5 **do**3:       Update the number of forgetting dimensions $k_{q}$ using Equation (29)4:       Initialize $X^{t+1}$ using Equation (27)5:       $\left(K_{1},\ldots ,K_{k_{q}}\right)\leftarrow \mathrm{randperm}\left(k_{q},N\right)$6:       **for** $i=\left(\left(q-1\right)\frac{N}{5}+1\right):\left(q\frac{N}{5}\right)$ **do**7:          **if** rand<u **then**8:             **Exploration:** update $x_{i,j}$ using Equation (28)9:          **else**10:            **Exploitation:** update $x_{i,j}$ using Equation (32)11:         **end if**12:      **end for**13:   **end for**14:**else**                 ▹ Pure exploitation stage15:   Update the number of forgetting dimensions $k_{r}$ using Equation (33)16:   Initialize $X^{t+1}$ using Equation (27)17:   $\left(K_{1},\ldots ,K_{k_{r}}\right)\leftarrow \mathrm{randperm}\left(k_{r},N\right)$18:   **for** i=1:N **do**19:      **Exploitation:** update $x_{i,j}$ using Equation (32)20:      Check bounds21:   **end for**22:**end if**23:**return** $X^{t+1}$

Once the continuous core of DOA is defined, additional phases are incorporated to enable its application to the SCP. First, a discretization stage is applied to transform continuous candidate solutions into binary representations.

### 6.2. Binary DOA Discretization Process

The Binary DOA (BDOA), Algorithm 4, takes a continuous population generated by the DOA core as input and applies the V3-ELIT discretization scheme to each dimension in order to obtain.
**Algorithm 4** Binary DOA using a V3-ELIT discretization scheme.**Input:** SCP instance (A,c), population size *N*, maximum iterations $T_{\max}$, DOA parameters (u,α), discretization scheme V3-ELIT, repair operator set R, AOS (MAB) parameter ε.**Output:** Best feasible binary solution $b^{\mathrm{best}}$.1: Initialize continuous population $X^{0}$ using Equations (24) and (25)2: t←03: **while** $t<T_{\max}$ **do**4:    Generate $X^{t+1}$ from $X^{t}$ using the DOA core (Algorithm 3)5:    Binarize $X^{t+1}$ using the V3-ELIT scheme6:    **if** $X^{t+1}$ is infeasible **then**7:       Select and apply repair operator from R using AOS8:    **end if**9:    Evaluate all feasible solutions in $X^{t+1}$10:   Update $b^{\mathrm{best}}$ if a better solution is found11:   t←t+112:**end while**13:**return** $b^{\mathrm{best}}$

**Figure 2:**
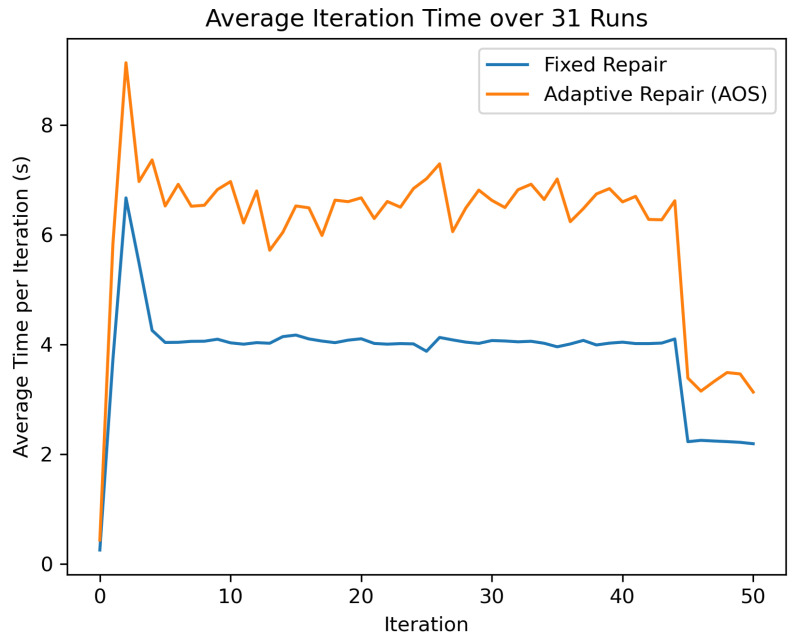
Average per iteration runtime across 31 runs comparing fixed repair and adaptive repair (AOS). The curves remain nearly parallel throughout the search, indicating a constant-factor overhead (≈60%) associated with adaptive repair rather than a change in asymptotic computational complexity.

The logic of the Algorithm 4 balances solution quality and computational overhead by activating repair operations only when infeasible solutions are generated (lines 6–8), thus avoiding unnecessary computational effort on solutions that are already feasible. At each iteration, a single repair operator is selected from a predefined set using AOS, rather than applying multiple repair strategies sequentially see the Figure 2.

Empirical analysis shows that the adaptive repair mechanism increases the average iteration time by approximately 60% compared to the fixed repair strategy (Wilcoxon test, $p\approx 5.0\times 10^{-5}$). The iteration time curves, which are practically parallel, indicate that this increase corresponds to a constant-factor overhead rather than a progressive growth in computational cost.

In exchange for this increase in execution time, the adaptive strategy achieves a statistically significant improvement in the average solution quality (mean improvement ≈1.65 cost units, Wilcoxon test $p\approx 3.97\times 10^{-5}$), shifting the distribution of results toward lower-cost regions.

### 6.3. Binary DOA Using AOS

The final algorithm, Algorithm 5, combines the continuous DOA core, the V3-ELIT binarization scheme, and an AOS module into a unified framework for solving the Set Covering Problem.
**Algorithm 5** Binary DOA with adaptive repair selection for the SCP.**Input:** SCP instance (A,c), population size *N*, maximum iterations $T_{\max}$, DOA parameters (u,α), discretization scheme V3-ELIT, repair operator set R, AOS (MAB) parameters (ε).**Output:** Best feasible binary solution $b^{\mathrm{best}}$.1: Initialize continuous population $X^{1}$ using Equations (24) and (25)2: Initialize AOS bandit estimates Q(a), N(a) for all a∈R3: $b^{\mathrm{best}}\leftarrow Ø$, $f^{\mathrm{best}}\leftarrow +\infty$4: t←05: **while** $t\leq T_{\max}$ **do**6:    Generate $X^{t+1}$ from $X^{t}$ using the DOA core, using Algorithm 37:    Binarize $X^{t+1}$ using the V3-ELIT scheme8:    **if** $X^{t+1}$ is infeasible **then**9:        Select repair operator $a_{t}$ using the AOS mechanism using Equation (21)10:      Apply the selected repair operator11:   **end if**                  ▹ AOS learning: reward computation12:   Evaluate all feasible solutions13:   Update $b^{best}$ and $f^{best}$14:   Compute reward $r_{t}$ using Equation (22)15:   Update bandit estimates $Q(a_{t})$ using Equation (23)16:   t←t+117:**end while**18:**return** $b^{\mathrm{best}}$

**Figure 3:**
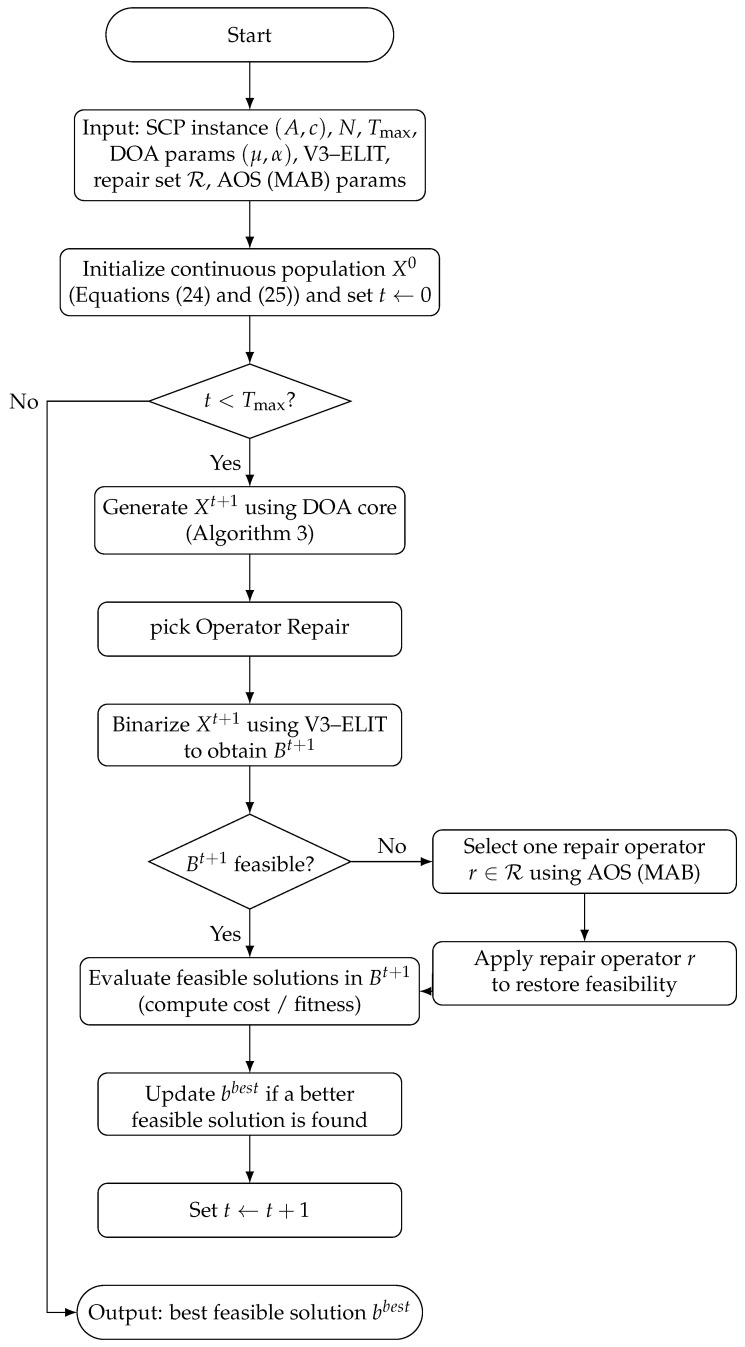
Flowchart of the proposed Binary DOA using V3-ELIT discretization and AOS. Repair is triggered only when infeasible solutions are produced; otherwise, the algorithm evaluates feasible solutions, updates the best solution, and iterates until $T_{\max}$.

To facilitate understanding of the proposed method, a flowchart representation of Algorithm 4 is provided in Figure 3. The diagram summarizes the main stages of the Binary DOA framework, including population initialization, continuous update through the DOA core, V3-ELIT discretization, feasibility verification, adaptive repair selection using AOS, solution evaluation, and best-solution update. This visual representation highlights the interaction between discretization and adaptive repair, as well as the conditional activation of the repair mechanism when infeasible solutions are generated.

## 7. Experimental Results

This section reports the experimental results obtained with the binary version of the DOA applied to the SCP. The analysis focuses on evaluating the impact of the feasibility restoration process and the adaptive selection of repair operators on solution quality and search stability. Results are presented using well known benchmark instances and are compared against established metaheuristic approaches to provide an objective and reproducible assessment.

### 7.1. Methodology

The experimental study was conducted using the solver described in Algorithm 4 and a set of classical SCP benchmark instances from the OR-Library, originally introduced by Beasley. The characteristics of these instances are summarized in Table 5 and include the sets 4, 5, 6, A, B, C, D, NRE, NRF, NRG, and NRH, which cover a wide range of problem sizes, coverage densities, and cost structures.

For each instance and experimental configuration, 31 independent runs were performed. In all experiments, a fixed maximum number of iterations was adopted as the stopping criterion, ensuring a homogeneous computational budget in terms of fitness evaluations for all compared configurations.

Performance was evaluated using quality and stability indicators commonly reported in the SCP literature, including best and median solution cost, relative percentage deviation (RPD) with respect to known optima, and the coefficient of variation (CV) across independent runs. To further analyze the behavior of the search process, additional indicators related to stagnation were considered, and non-parametric statistical tests were employed to support the comparative assessment.

For the study and evaluation of the metaheuristic, we relied on the evaluation metrics and methodologies used in a fairly recent study, due to their demonstrated effectiveness in providing a solid framework to ground and compare the results objectively. Including the following:

- Relative Percentage Deviation (RPD): Quantifies the quality of a solution *x* by comparing it to the known optimal value $\hat{x}$. Measuring the percentage difference.
  (34) $$RPD=\frac{x-\hat{x}}{\hat{x}}\times 100$$
- Coefficient of Variation (CV): Evaluates the stability of an algorithm’s solutions by measuring the ratio of the standard deviation σ to the mean μ, a lower CV means less variation.
  (35) $$\mathrm{CV}=\frac{\sigma }{\mu }\times 100$$

Lastly, the results were put into a statistical evaluation to objectively assess the overall performance of both the DOA and the metaheuristics to which we will compare. This will ultimately ensure that the data is normalized and allowed for fair and consistent comparisons.

### 7.2. Parameter Settings

To define the experimental parameters, a preliminary tuning phase was conducted using one representative instance from each problem family. This procedure is necessary because the performance of metaheuristics is highly sensitive to their control parameters, and an inadequate configuration can distort the comparison between algorithms or introduce artificial biases in the results. In this exploratory phase, the population size was set to 100 individuals, the maximum number of iterations to 50, and a single stochastic greedy repair operator was used within the AOS framework. The results of this phase are shown Table 6 and Table 7.

### 7.3. Sensitivity Analysis of the Transition Parameter α

The parameter α in Equation (24) defines the transition point between the mixed exploration and exploitation phase and the pure exploitation phase. Since this parameter directly influences the search dynamics, its impact on performance was evaluated through a sensitivity analysis.

To evaluate whether the transition parameter α significantly influences the performance of the proposed algorithm across different problem sizes, a sensitivity analysis was conducted on 15 SCP benchmark using 31 independent runs for each α∈{0.6,0.7,0.8,0.9}. For each configuration, the median fitness value per instance was computed (Table 8). A global comparison using the Friedman test (Table 9) yielded p=0.160, indicating that no statistically significant differences exist among the tested values.

These results suggest that the proposed method is robust with respect to moderate variations in α. Therefore, α=0.9 was retained for the main experiments, as it maintains a longer mixed exploration and exploitation phase without degrading performance.

Although α=0.8 achieved the best average ranking, the Friedman test indicated no statistically significant differences among the tested configurations (p=0.160). Given the absence of significant performance differences, the selection of α=0.9 was motivated by methodological considerations. A higher α value delays the transition to the pure exploitation phase, allowing for a longer mixed exploration–exploitation behavior, which aligns with the exploratory nature of the proposed algorithm. Since no degradation in performance was observed, α=0.9 was retained as a stable and conceptually consistent choice.

Finally, the configuration adopted for the experimental study was defined with the objective of achieving high-quality results. These settings are reported in the Table 10.

The experiments were executed on

- Processor: AMD Ryzen AI 9 HX 370 with Radeon 890M (2.00 GHz)
- Installed RAM: 32.0 GB (31.1 GB usable)
- System type: 64-bit operating system, x64-based processor

### 7.4. Performance Analysis

**Figure 4:**
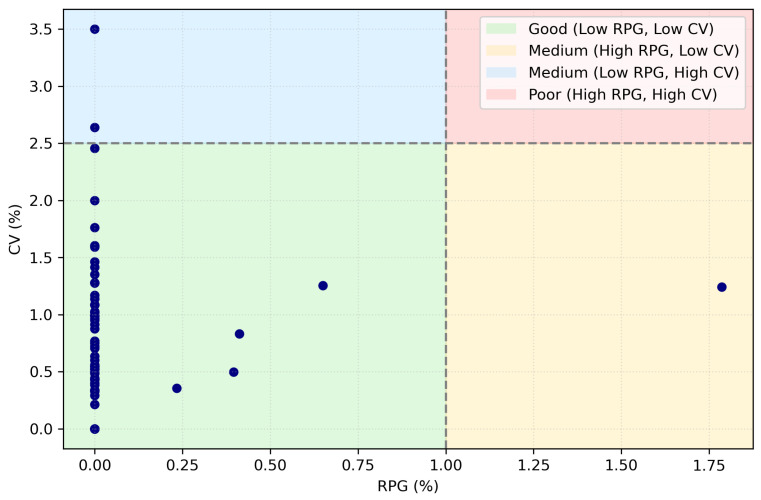
Scatterplots, RPD vs. CV for each binarization method performance.

The experimental results exhibit robust and stable performance, as can be observed in the quality zone plot [36] shown in Figure 4. Undoubtedly, the AOS mechanism used to select the repair operator significantly improves the search process by dynamically choosing the most appropriate repair strategy according to the observed behavior of the algorithm. In the following, we present the results of our experiments in the Table 11.

The experimental results indicate that our algorithm achieves a high proportion of optimal or near-optimal solutions across the evaluated instances. In particular, approximately 92.3% of the instances attained an RPD value equal to zero, while the remaining cases exhibited RPD values below one, suggesting a low deviation from the known optima. From a structural perspective, the adaptive repair selection mechanism is activated only under stagnation or feasibility violations, enabling the diversification of the search when required.

From the point of view of process structure, the AOS mechanism responsible for selecting the repair process works efficiently by adaptively activating diversified repair operators only when required by the search dynamics of the algorithm, especially in situations of stagnation or feasibility violations. The consistently low values obtained for the standard deviation and coefficient of variation further confirm the robustness and stability of the proposed approach in different cases.

The Figure 4 graph allows simultaneous evaluation of quality (RPD%) and robustness (CV%). In our case, with RPD = 1.0% and CV = 2.5%, the vast majority of results are located in the green region (low RPD, low CV), with 57 out of 60 points located in this quadrant, which shows consistent and stable behavior in most instances. Only three cases are outside this range: nrf3 and nrf4 show high variability (CV ≈ 3.50% and 2.64%, respectively), while nrg4 corresponds to a case of quality degradation (RPD ≈ 1.79%), indicating specific instances where the method is more sensitive and which, therefore, are natural candidates for specific analysis and adjustment.

### 7.5. General Analysis Repair Strategies Method

This subsection reports the performance indicators associated with the repair operators used in the proposed SCP solver. The analysis is based on the activation frequency, cumulative and average reward, mean fitness improvement (mean delta), and success rate of each operator. These metrics are not intended to evaluate the repair operators as standalone optimization methods, but rather to quantify their contribution within the adaptive selection mechanism.

By analyzing how often each operator is selected and how its activations are associated with changes in solution quality, this subsection provides a quantitative description of the behavior of the adaptive repair mechanism during the search process. Table 3 summarizes the repair strategies considered in this study, while Table 12 defines the metrics and notation used to characterize the behavior of the repair operators, and Table 13 reports the per-instance operator statistics.

The activation patterns observed (Table 13) in repair operators are consistent with the quality indicators obtained from the experimental results, especially concerning the occurrence of solutions with zero RPD. The predominance of complex stochastic repair, which accounts for 18,088 activations (46.4% of the total), coincides with a higher incidence of executions reaching an RPD = 0, indicating that this operator frequently participates in trajectories that reach known optimal solutions or best known solutions. Complex penalized repair, despite being activated 11,090 times (28.4%), is mainly associated with stable but less transformative search behavior, which limits its contribution to obtaining RPD=0. The complex stochastic operator, with 9851 activations (25.2%), shows intermediate behavior, favoring occasional improvements but with less impact on the frequency of RPD = 0 results. The basic complex repair operator was never activated by the adaptive mechanism, indicating that it consistently received the lowest expected reward among all strategies.

**Figure 5:**
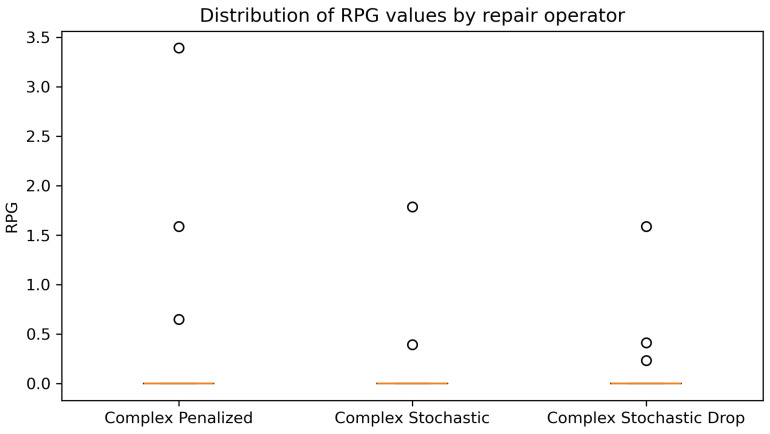
Boxplot of final RPD values by repair operator. The low median and reduced interquartile range indicate consistently high solution quality and robust performance across independent runs, with only occasional deviations from optimality.

The Figure 5 representation highlights both the quality and robustness of the process architecture. The boxes are concentrated near RPD = 0 and show a very small interquartile range, demonstrating that executions consistently achieve high quality solutions with low dispersion. The small size of the boxes reflects stable behavior in independent executions, while the limited presence of outliers suggests that deviations from optimization are infrequent. Table 14 presents the global performance comparison of the evaluated algorithms.

**Figure 6:**
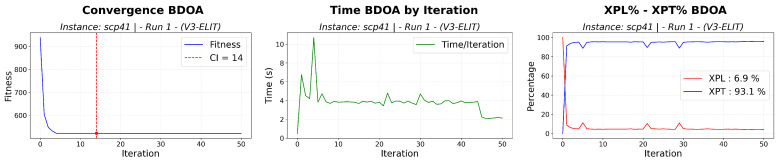
BDOA convergence, XPL-XPT balance and time (scp41 set).

**Figure 7:**
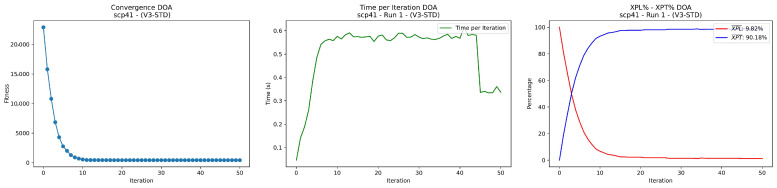
DOA convergence, XPL-XPT balance and time (scp41 set) without repair diversification.

The instance scp41 is selected as a representative case study, as it corresponds to a non-trivial scenario where the optimal solution is not consistently reached, allowing a meaningful analysis of the search dynamics and the contribution of repair operators. The localized fluctuations observed in the XPL and XPT into Figure 6 curves correspond to controlled diversification events triggered by stagnation or low improvement phases. The rapid recovery of exploitation dominance after these events indicates that exploratory interventions are effectively absorbed by the search process, preserving stability while enabling escape from local optima. The Figure 7 illustrate a predominantly exploitation driven search process, characterized by rapid convergence and limited exploratory activity, highlighting the baseline behavior of the algorithm when repair diversification is not employed.

### 7.6. Comparative Analysis

BDOA was evaluated alongside a set of highly regarded metaheuristics in the field of combinatorial optimization: SCA [38], PSA [39], GWO [40], and BGO [13]. The main features that make them attractive are: SCA is characterized by a lightweight formulation and a balanced exploration exploitation mechanism; PSA follows a physics-inspired search paradigm and has demonstrated competitive performance in discrete optimization contexts; GWO is a widely adopted approach in binary search spaces with stable convergence behavior; and BGO represents a more recent binary oriented variant designed to improve solution quality in discrete domains. All algorithms are evaluated using the best solution cost obtained in reference SCP instances, ensuring a consistent and reproducible evaluation framework. The experimental results have been extracted from previously published data and are summarized in Table 15. The results for SCA, PSA, GWO, and BGO were obtained from experiments alternating combinations of DS and with a fixed repair operator.

**Figure 8:**
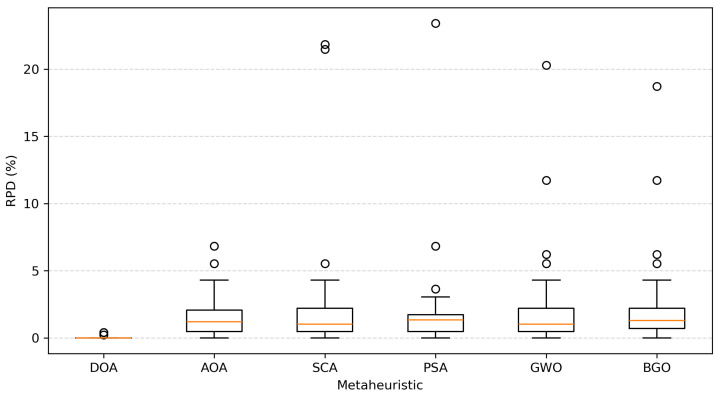
Boxplot of RPD values across metaheuristics over 45 SCP instances, illustrating solution quality and robustness.

The results shown in Table 13 provide clear evidence of DOA’s superior performance in the 45 SCP instances. DOA achieves the lowest average minimum RPD (0.02), indicating near optimal solution quality on average, and obtains the best result in 42 of the 45 instances. This dominance is reinforced by an average ranking of 1.00, confirming that DOA consistently outperforms competing metaheuristics across the benchmark. A boxplot representation of RPD values is included to visualize the distribution, robustness, and variability of each metaheuristic across instances; see Figure 8.

In addition, a normality analysis was conducted on the 31 independent executions of DOA for each SCP instance. The results reveal a predominantly non-Gaussian behavior of the performance distributions, supporting the use of median-based indicators and interquartile ranges to characterize robustness. This analysis is intended solely to describe the internal performance distribution of DOA and does not constitute a statistical comparison with competing algorithms. The results are in Table 16 and Table 17.

### 7.7. Statistical Analysis

The Table 18 presents the results of the Wilcoxon signed-rank test applied to the RPD values obtained across all instances of the SCP, comparing the DOA algorithm against each reference metaheuristic.

The comparison column indicates the pairwise comparison performed between DOA and each competing algorithm (AOA, SCA, PSA, GWO, and BGO). Each comparison is carried out on an instance by instance basis using the corresponding RPD values.

The wins column represents the number of instances in which DOA achieved a lower RPD than the compared algorithm. The ties column indicates the number of instances in which both algorithms achieved exactly the same RPD value, meaning equivalent performance. The losses column shows the number of instances in which DOA obtained a higher RPD than the compared algorithm. Finally, the *p*-value column corresponds to the statistical significance value obtained from the Wilcoxon test. This value tests whether the median difference between the two algorithms is zero. Since all *p*-values are below α = 0.05, the differences are statistically significant, indicating that DOA performs significantly better than all compared metaheuristics.

The obtained Wilcoxon test results (*p*-values on the order of $10^{-8}$) indicate that the observed differences between DOA and each of the compared metaheuristics are not due to random variation. Since all *p*-values are significantly below the significance level α=0.05, the null hypothesis is rejected in every case. Furthermore, the wins–ties–losses analysis reinforces this evidence: DOA presents no losses against any algorithm and exhibits a dominant number of wins across the evaluated instances.

Overall, these results allow us to conclude that DOA demonstrates statistically significant, consistent, and robust superior performance over the complete set of considered SCP instances.

Finally, Table 19 presents the average ranking results together with the corresponding effect size analysis based on Cliff’s delta. The average rank column reports the mean rank obtained by each algorithm across all evaluated SCP instances, where lower values indicate better overall performance. As observed, DOA achieves the lowest average rank, confirming its consistent superiority across the benchmark set.

To complement the ranking analysis, Cliff’s delta was computed between DOA and each competing metaheuristic in order to quantify the magnitude of the performance differences. All reported effect sizes are above 0.80, which corresponds to a very large effect according to established interpretation thresholds. This indicates that the superiority of DOA is not only reflected in its ranking position but is also substantial in practical terms. Together, these results provide robust statistical evidence supporting the dominance of DOA over the compared algorithms.

## 8. Novelty and Contributions

The main contribution of this work is the incorporation of a complementary learning assisted repair mechanism within the DOA to address the SCP. Rather than modifying the core search dynamics of the metaheuristic, the proposed mechanism operates as an auxiliary decision support component that actively guides candidate solutions toward feasible and high quality regions of the search space. The experimental results show that this hybrid framework is able to consistently reach known optimal solutions across multiple SCP instances, indicating a clear improvement in solution refinement capabilities when compared to the standalone behavior typically observed in discretization-based approaches.

A distinctive aspect of the proposed methodology is that the learning-based repair component is architecturally decoupled from DOA. The mechanism is not designed as a problem specific or algorithm specific operator, but rather as a modular and portable tool that can be integrated into other metaheuristics employing binary representations or repair-based feasibility restoration. This design choice enables the reuse of the proposed architecture in different optimization frameworks, supporting its applicability beyond the specific case of DOA and SCP.

From a methodological perspective, the proposed framework was evaluated through 31 independent executions per instance, allowing a rigorous characterization of robustness and consistency. The observed convergence to optimal solutions, together with the non-Gaussian and highly concentrated performance distributions, supports the role of the complementary repair mechanism as an effective enhancer of solution quality without increasing the algorithmic complexity of the underlying metaheuristic.

Overall, this work contributes a portable and extensible hybrid optimization architecture in which a lightweight learning assisted repair mechanism complements a metaheuristic search process, improving its ability to reach high quality and optimal solutions in combinatorial optimization problems.

Finally, the adaptive repair selection mechanism evaluated in this study can be viewed as a general decision component that can be integrated into other coverage-based optimization problems beyond the Set Covering Problem.

## 9. Conclusions

This paper addressed the SCP using an adapted version of the DOA, complemented by a learning assisted repair mechanism. The proposal does not alter the fundamental logic of the metaheuristic but introduces an auxiliary component that intervenes during the repair process with the aim of improving the feasibility and quality of the solutions generated. This approach allowed the original structure of the algorithm to be maintained, while reinforcing its ability to perform in a discrete and highly restricted search space.

The results obtained show that the proposed scheme is capable of repeatedly achieving known optimal solutions in a wide range of SCP instances. This consistent convergence, observed across multiple independent runs, highlights the usefulness of the repair mechanism in supporting the search process, facilitating the transition of candidate solutions to high quality feasible regions. Likewise, analysis of the internal behavior of the algorithm reveals stable dynamics, characterized by low dispersion of results and a marked concentration around optimal solutions, which reinforces the reliability of the approach adopted.

The descriptive comparison with other widely used metaheuristics indicates that DOA performs competitively in terms of solution quality on the benchmarks considered. Although no inferential statistical comparisons between algorithms were performed due to the limited availability of independent runs for the comparative methods, the analysis presented offers a clear and methodologically consistent view of the relative positioning of the proposed approach. Overall, the results suggest that combining DOA with a learning assisted repair mechanism is an effective and flexible alternative for solving combinatorial optimization problems.

## 10. Future Works

The results obtained in this study generate several possibilities for further study and can be addressed in subsequent research. Mainly, it is pertinent to examine in greater detail the specific contribution of the learning assisted repair mechanism through comparisons made under strictly equivalent algorithmic configurations. This point is important because it generates a line of research to test new repair operators and, on the other hand, to seek other learning mechanisms.

On the other hand, the proposed architecture was designed without a rigid coupling to DOA, which facilitates its incorporation into other metaheuristic schemes aimed at binary or combinatorial optimization. Exploring its integration into different algorithms would allow evaluating its degree of generality and determining whether the benefits observed in this work are maintained in different algorithmic contexts.

Finally, it would be interesting to extend the evaluation to larger instances or those with characteristics closer to real applications, where the constraints and scale of the problem impose additional challenges on the search process and the time cost of the process. Such scenarios would allow for a more accurate assessment of the practical scope of the proposed approach and delimit its possible limitations.

## Figures and Tables

**Table 1:** Illustrative example of an SCP formulation for a marketing application.

Medium *j*	Segments Covered $c_{j}$	Cost $c_{j}$
National TV	Youth, Adults, Families	100
Radio	Adults, Elderly	40
Professional Magazine	Professionals, Adults	60
Instagram	Youth, Professionals	50
Facebook	Youth, Adults, Families, Elderly	70
Daily	Adults, Families	30

**Table 2:** Conceptual classification of repair operators in metaheuristics and their roles in feasibility restoration and search control.

Repair Category	Conceptual Role
Corrective repair	Corrective repair operators aim to restore feasibility without explicitly considering their impact on solution quality. They typically act in a deterministic or greedy manner by activating the minimum components required to satisfy violated constraints, often by adding columns to cover uncovered elements without evaluating the induced cost increase. Although feasibility is ensured, these operators tend to introduce structural bias and may quickly lead to overloaded solutions or search plateaus, limiting further improvement [9,14].
Guided repair	Corrective repair operators aim solely to restore the feasibility of infeasible solutions without explicitly considering their impact on solution quality. They typically act deterministically by activating the minimum components required to satisfy violated constraints, adding columns to cover uncovered elements without evaluating the induced cost increase. Although feasibility is guaranteed, these operators often introduce structural bias and may quickly lead to overloaded solutions or search plateaus, limiting further improvement [9,14].
Diversifying repair	Diversifying repair operators introduce stochastic mechanisms or structural perturbations to avoid repetitive repair patterns and mitigate stagnation. Unlike corrective and guided approaches, these operators actively seek to alter the solution structure through probabilistic selection of columns, controlled perturbations, or partial removal of redundant components. In the SCP and other constrained combinatorial problems, they have been shown to be effective in escaping plateaus and local optima induced by discretization and deterministic repair processes [25,32].
Hybrid repair	Hybrid repair operators combine feasibility restoration with solution refinement, treating repair as an active operator with a direct impact on solution quality. In the SCP, they typically incorporate add–drop mechanisms, penalization of redundant columns, or incremental cost evaluation during repair. This approach aligns with adaptive metaheuristic and operator selection frameworks, where repair functions as a high-level search operator whose activation can be dynamically learned [26,27].

**Table 3:** Functional categorization of the repair operator set used in the proposed approach.

Repair Operator	Category	Justification
Complex	Corrective repair	Deterministically restores feasibility by activating columns to satisfy uncovered constraints, following coverage-based strategies commonly reported in SCP literature [9,14].
Complex Penalized	Guided repair	Extends the deterministic strategy by incorporating cost- and penalty-aware selection rules to discourage redundant or expensive columns during feasibility restoration [34].
Complex Stochastic	Diversifying repair	Introduces controlled stochasticity by probabilistically selecting candidate columns, breaking deterministic repair patterns and promoting structural diversification [25,32].
Complex Stochastic Drop	Hybrid repair	Combines stochastic feasibility restoration with explicit removal of active columns (add–drop mechanism), followed by a re-repair phase to ensure feasibility and enable strong diversification [27].

**Table 4:** Notation and functional role of the terms used in the bandit reward mechanism.

Symbol	Name	Description and Role
*t*	Iteration index	Current iteration of the optimization process; defines the time step at which the bandit decision and reward update are performed.
$f_{\mathrm{best}}^{\left(t\right)}$	Global best fitness	Best objective function value found up to iteration *t*; represents the current search state and serves as the reference to evaluate whether a repair operator contributes to solution improvement.
$f_{\mathrm{best}}^{\left(t-1\right)}$	Previous global best fitness	Best objective value before applying the selected repair operator; used as a baseline to quantify the effect of the selected repair action.
$r_{t}$	Instantaneous reward	Absolute improvement in the global best solution, computed as $r_{t}=f_{\mathrm{best}}^{\left(t-1\right)}-f_{\mathrm{best}}^{\left(t\right)}$; provides the learning signal used to update the estimated value of the selected repair operator.
$a_{t}$	Selected repair operator	Repair operator chosen by the bandit mechanism at iteration *t*; represents the action whose performance is being evaluated and learned over time.
Q(a)	Estimated action value	Estimated expected reward of repair operator *a*; guides exploitation by indicating which operator has historically contributed most to search progress.
N(a)	Update counter	Number of times operator *a* has produced a positive reward; controls the learning rate in the incremental update rule.
ε	Exploration rate	Probability of selecting a random repair operator; ensures continued exploration of alternative strategies and prevents premature convergence to suboptimal operators.

**Table 5:** Description of the OR-Library SCP benchmark sets, including number of instances, problem size, cost range, density, and optimal solution status. Data taken from [36]).

Instance Family	Number of Instances	m	n	Cost Range	Density (%)	Optimal Solution
4	10	200	1000	[1, 100]	2.00	known
5	10	200	2000	[1, 100]	2.00	known
6	5	200	1000	[1, 100]	5.00	known
A	5	300	3000	[1, 100]	2.00	known
B	5	300	3000	[1, 100]	5.00	known
C	5	400	4000	[1, 100]	2.00	known
D	5	400	4000	[1, 100]	5.00	known
NRE	5	500	5000	[1, 100]	10.00	known
NRF	5	500	5000	[1, 100]	20.00	known
NRG	5	1000	10,000	[1, 100]	2.00	BKS (not proven)
NRH	5	1000	10,000	[1, 100]	5.00	BKS (not proven)

Note: For the NRG and NRH families, the reference values correspond to best known solutions (BKS), as optimality has not been formally proven for these instances.

**Table 6:** Results obtained in pre-setting phase.

Instance	Best	Min Fitness	RPD Min	Max Fitness	RPD Max	Min Time	Max Time
41	429	430	0.233100	434	1.165501	42.429	95.47
51	253	253	0	256	1.18577	64.689	168.627
61	138	138	0	143	3.623188	36.154	86.078
a1	253	254	0.39525	318	25.691699	93.727	303.665
b1	69	69	0	70	1.449275	94.149	205.881
c1	227	227	0	235	3.524229	142.251	313.151
d1	60	60	0	62	3.333333	122.494	378.911
nre1	29	29	0	50	72.413793	173.964	490.585
nrf1	14	14	0	15	7.142857	198.673	747.929
nrg1	176	176	0	187	6.25	699.833	1467.229
nrh1	63	64	1.587301	72	14.285714	671.997	2172.43

**Table 7:** Parameter settings configuration, preliminary testing.

Category	Parameter	Value
Global Settings	Population Size	500
	Iterations	25
	Number of Experiments	31
	Bounds	[0, 1]
	Binarization Methods	V3-ELIT
	Operator Repair used.	Complex Stochastic (Table 3)
DOA Settings	*u*	0.9
	α	0.9

**Table 8:** Median fitness values obtained for each α across 15 SCP instances (31 runs per configuration).

Instance	α=0.6	α=0.7	α=0.8	α=0.9
scp41	433	432	432	433
scp42	514	513	513	513
scp43	523	520	521	523
scp44	495	498	495	495
scp45	515	514	514	514
scp51	256	258	257	257
scp52	312	309	310	310
scp53	228	229	228	229
scp54	245	244	244	243
scp55	212	212	212	212
scp61	140	140	140	138
scp62	148	148	147	147
scp63	145	148	145	148
scp64	131	132	131	131
scp65	163	162	161	164

**Table 9:** Global comparison of α values using Friedman test.

α	Average Rank	Interpretation
0.8	2.000	Best rank
0.9	2.467	Second
0.7	2.667	Third
0.6	2.867	Fourth
Friedman test *p*-value = 0.160		

**Table 10:** Parameter settings configuration.

Category	Parameter	Value
Global Settings	Population Size	500
	Iterations	31
	Number of Experiments	31
	Bounds	[0, 1]
	Binarization Methods	V3-ELIT
	Repair operator set	The set is defined in Table 3.
DOA Settings	*u*	0.9
	α	0.9

**Table 11:** Unified statistical summary of DOA experimental results across all SCP instances, including solution quality, variability, RPD, and computational performance metrics. RPD and CV are presented in percentages, and mean time is presented in seconds.

Inst	Opt	Best	Max	Avg	Std	RPD	Mean Time	CV
41	429	430	434		1.5268	0.2331	312.8115	0.3540
42	512	512	519	513.0645	1.7500	0.0000	319.2201	0.3411
43	516	516	541	519.8065	5.6534	0.0000	289.5999	1.0876
44	494	494	510	495.5806	3.1493	0.0000	279.7354	0.6355
45	512	512	523	514.0968	2.2264	0.0000	271.0674	0.4331
46	560	560	568	561.9677	2.1523	0.0000	280.3740	0.3830
47	430	430	443		2.4097	0.0000	277.5383	0.5593
48	492	492	501	493.3548	1.6643	0.0000	250.4862	0.3373
49	641	641	673	647.3548	6.6460	0.0000	304.8988	1.0266
410	514	514	524	514.7742	2.0609	0.0000	327.8392	0.4004
51	253	253	263	254.6452	2.2293	0.0000	392.1783	0.8755
510	265	265	271	265.4839	1.3873	0.0000	335.2115	0.5226
52	302	302	313	305.4194	3.3144	0.0000	371.7686	1.0852
53	226	226	230	228.0968	0.9783	0.0000	295.8237	0.4289
54	242	242	246	242.7097	1.2700	0.0000	346.7195	0.5233
55	211	211	216	211.7742	1.0555	0.0000	334.8404	0.4984
56	213	213	213	213.0000	0.0000	0.0000	328.3905	0.0000
57	293	293	295	293.5161	0.6256	0.0000	329.1240	0.2131
58	288	288	296	288.6774	1.5788	0.0000	326.6231	0.5469
59	279	279	287	280.1290	1.3599	0.0000	291.1548	0.4855
61	138	138	143	138.9677	1.5808	0.0000	109.2588	1.1375
62	146	146	150	147.0000	1.0646	0.0000	111.0327	0.7242
63	145	145	148	145.7742	1.3344	0.0000	121.4858	0.9154
64	131	131	131	131.0000	0.0000	0.0000	116.1451	0.0000
65	161	161	169	162.0645	1.8962	0.0000	109.7083	1.1700
a1	253	254	259	256.1935	1.2759	0.3953	312.9886	0.4980
a2	252	252	256	252.9355	1.1236	0.0000	326.0005	0.4442
a3	232	232	240	235.0000	2.3944	0.0000	499.1275	1.0189
a4	234	234	241	236.0645	1.7689	0.0000	539.9109	0.7493
a5	236	236	245	237.9032	1.8322	0.0000	531.1673	0.7701
b1	69	69	74	69.2903	0.9379	0.0000	259.6743	1.3535
b2	76	76	77	76.0645	0.2497	0.0000	316.7963	0.3283
b3	80	80	81	80.6452	0.4864	0.0000	251.2323	0.6031
b4	79	79	81	79.2258	0.5603	0.0000	248.3629	0.7073
b5	72	72	72	72.0000	0.0000	0.0000	213.3265	0.0000
c1	227	227	235	230.0645	2.1899	0.0000	399.2950	0.9519
c2	219	219	227	222.0000	2.1448	0.0000	423.4509	0.9661
c3	243	244	252	245.6452	2.0420	0.4115	461.3300	0.8313
c4	219	219	228	221.6129	2.2012	0.0000	399.0985	0.9933
c5	215	215	219	216.1290	1.1759	0.0000	484.2342	0.5441
d1	60	60	64	60.9032	0.9702	0.0000	1244.3470	1.5930
d2	66	66	69	66.7742	0.6633	0.0000	1228.9727	0.9934
d3	72	72	75	72.9355	0.7214	0.0000	1207.7612	0.9890
d4	62	62	62	62.0000	0.0000	0.0000	1424.6705	0.0000
d5	61	61	62	61.0323	0.1781	0.0000	1248.9425	0.2919
nre1	29	29	29	29.0000	0.0000	0.0000	672.2805	0.0000
nre2	30	30	32	30.6774	0.5408	0.0000	586.0562	1.7629
nre3	27	27	28	27.7419	0.4448	0.0000	640.5682	1.6034
nre4	28	28	30	28.4839	0.5699	0.0000	647.9322	2.0006
nre5	28	28	28	28.0000	0.0000	0.0000	614.2474	0.0000
nrf1	14	14	15	14.0323	0.1796	0.0000	1025.3873	1.2799
nrf2	15	15	15	15.0000	0.0000	0.0000	1171.4226	0.0000
nrf3	14	14	15	14.5161	0.5080	0.0000	1140.6485	3.4996
nrf4	14	14	15	14.1613	0.3739	0.0000	1075.3477	2.6401
nrf5	13	13	14	13.8710	0.3408	0.0000	811.6981	2.4568
nrg1	176	176	185	178.8387	2.2818	0.0000	2484.3525	1.2759
nrg2	154	155	163	157.9355	1.9822	0.6494	3463.8843	1.2551
nrg3	166	166	176	170.2581	2.4895	0.0000	3708.3079	1.4622
nrg4	168	171	180	173.6129	2.1553	1.7857	4497.9010	1.2414
nrg5	168	168	177	170.7742	2.4181	0.0000	4580.0534	1.4160

**Table 12:** Definition of repair operator performance metrics.

Metric	Description
Inst	SCP instance identifier.
Operator Repair	Repair operator selected by the adaptive mechanism.
Total	Cumulative reward obtained by the operator over all its activations for the instance.
Mean	Average reward per activation of the operator.
Activations	Number of times the operator was selected by the adaptive mechanism.
Mean delta	Mean improvement in solution cost after applying the repair operator.
Success rate	Proportion of activations that resulted in an improvement of the solution cost.

**Table 13:** Per-instance repair operator performance summary (values rounded to four decimals).

Inst	Operator Repair	Total	Mean	Activations	Mean Delta	Success Rate
scp41	complex_stochastic_drop	46,813	84.8062	552	84.8062	0.1467
scp42	complex_penalized	38,677	77.3540	500	77.3540	0.0440
scp43	complex_penalized	37,440	68.1967	549	68.1967	0.0364
scp44	complex_penalized	39,956	75.2467	531	75.2467	0.0377
scp45	complex_penalized	32,546	78.2356	416	78.2356	0.0577
scp46	complex_stochastic_drop	46,418	77.8826	596	77.8826	0.1762
scp47	complex_stochastic_drop	35,100	50.0713	701	50.0713	0.1384
scp48	complex_stochastic_drop	44,224	72.4984	610	72.4984	0.1197
scp49	complex_stochastic_drop	49,872	77.2012	646	77.2012	0.1889
scp410	complex_stochastic	49,478	74.8533	661	74.8533	0.0908
scp51	complex_stochastic	105,134	161.7446	650	161.7446	0.1123
scp510	complex_stochastic_drop	37,880	326.5517	116	326.5517	0.1034
scp52	complex_stochastic_drop	111,528	198.4484	562	198.4484	0.2616
scp53	complex_stochastic	140,655	192.1516	732	192.1516	0.0751
scp54	complex_stochastic_drop	88,129	145.4274	606	145.4274	0.1403
scp55	complex_stochastic_drop	133,198	250.8437	531	250.8437	0.1431
scp56	complex_stochastic_drop	145,425	252.9130	575	252.9130	0.1426
scp57	complex_stochastic_drop	97,451	173.0924	563	173.0924	0.1581
scp58	complex_stochastic_drop	128,694	258.9416	497	258.9416	0.1710
scp59	complex_penalized	97,917	187.5805	522	187.5805	0.0536
scp61	complex_stochastic	56,460	82.3032	686	82.3032	0.0743
scp62	complex_stochastic_drop	49,100	110.8352	443	110.8352	0.1219
scp63	complex_stochastic_drop	54,791	84.2938	650	84.2938	0.0892
scp64	complex_penalized	58,570	93.1161	629	93.1161	0.0556
scp65	complex_penalized	68,598	106.6843	643	106.6843	0.0886
scpa1	complex_stochastic	184,993	288.1511	642	288.1511	0.0639
scpa2	complex_stochastic_drop	209,912	386.5783	543	386.5783	0.1786
scpa3	complex_stochastic_drop	177,828	328.7024	541	328.7024	0.1922
scpa4	complex_penalized	262,349	328.7581	798	328.7581	0.0388
scpa5	complex_stochastic	195,225	317.4390	615	317.4390	0.0829
scpb1	complex_stochastic	250,357	419.3585	597	419.3585	0.0905
scpb2	complex_stochastic_drop	386,071	509.3285	758	509.3285	0.0923
scpb3	complex_stochastic	311,748	473.0622	659	473.0622	0.0956
scpb4	complex_stochastic_drop	246,465	463.2801	532	463.2801	0.1278
scpb5	complex_stochastic	256,868	456.2487	563	456.2487	0.0959
scpc1	complex_penalized	369,251	547.0385	675	547.0385	0.0459
scpc2	complex_penalized	371,908	579.2960	642	579.2960	0.0467
scpc3	complex_stochastic_drop	385,720	658.2253	586	658.2253	0.2082
scpc4	complex_stochastic	416,856	622.1731	670	622.1731	0.1358
scpc5	complex_stochastic_drop	455,536	683.9880	666	683.9880	0.1862
scpd1	complex_stochastic_drop	763,518	658.2052	1160	658.2052	0.1328
scpd2	complex_stochastic_drop	653,540	685.0524	954	685.0524	0.1247
scpd3	complex_stochastic_drop	343,187	659.9750	520	659.9750	0.1481
scpd4	complex_stochastic_drop	500,385	636.6221	786	636.6221	0.0738
scpd5	complex_stochastic_drop	372,450	665.0893	560	665.0893	0.1054
scpnre1	complex_stochastic	455,532	856.2632	532	856.2632	0.0959
scpnre2	complex_penalized	461,615	768.0782	601	768.0782	0.0782
scpnre3	complex_stochastic_drop	410,755	776.4745	529	776.4745	0.0870
scpnre4	complex_stochastic	499,095	802.4035	622	802.4035	0.0932
scpnre5	complex_penalized	502,046	831.2020	604	831.2020	0.0728
scpnrf1	complex_penalized	493,381	761.3904	648	761.3904	0.0648
scpnrf2	complex_penalized	479,606	778.5812	616	778.5812	0.0552
scpnrf3	complex_stochastic_drop	409,492	756.9168	541	756.9168	0.1201
scpnrf4	complex_penalized	439,935	831.6352	529	831.6352	0.0851
scpnrf5	complex_stochastic	407,694	828.6463	492	828.6463	0.0793
scpnrg1	complex_stochastic	980,110	1604.1080	611	1604.1080	0.1195
scpnrg2	complex_penalized	1,010,055	1368.6382	738	1368.6382	0.0515
scpnrg3	complex_stochastic	945,770	1798.0418	526	1798.0418	0.1521
scpnrg4	complex_stochastic	948,514	1652.4634	574	1652.4634	0.1359
scpnrg5	complex_stochastic_drop	1,472,335	1873.1997	786	1873.1997	0.1807
scpnrh1	complex_penalized	1,057,816	1425.6280	742	1425.6280	0.0512
scpnrh2	complex_stochastic_drop	1,009,107	1994.2826	506	1994.2826	0.1937
scpnrh3	complex_penalized	1,118,686	1582.2999	707	1582.2999	0.0537
scpnrh4	complex_stochastic_drop	949,810	2012.3093	472	2012.3093	0.2076
scpnrh5	complex_stochastic	66,885	3520.2632	19	3520.2632	0.2632

**Table 14:** Participation of repair operators per SCP instance achieving RPD = 0. Checkmarks indicate instances with RPD = 0. For instances where the minimum RPD is not zero, the corresponding RPD value is reported.

Inst	Complex	Complex Penalized	Complex Stochastic	Stochastic Drop
scp41		0.23		
scp42		✓		
scp43		✓		
scp44		✓		
scp45		✓		
scp46				✓
scp47				✓
scp48				✓
scp49				✓
scp410			✓	
scp51			✓	
scp52				✓
scp53			✓	
scp54			✓	
scp55			✓	
scp56		✓		
scp57				✓
scp58			✓	
scp59			✓	
scpa1		0.3953		
scpa2				✓
scpa3				✓
scpa4				✓
scpa5				✓
scpb1				✓
scpb2				✓
scpb3				✓
scpb4				✓
scpb5				✓
scpc1				✓
scpc2				✓
scpc3		0.4115		
scpc4				✓
scpc5				✓
scpd1				✓
scpd2				✓
scpd3				✓
scpd4				✓
scpd5				✓
scpnre1		✓		
scpnre2				✓
scpnre3		✓		
scpnre4				✓
scpnre5		✓		
scpnrf1				✓
scpnrf2				✓
scpnrf3				✓
scpnrf4				✓
scpnrf5				✓
scpnrg1				✓
scpnrg2		0.6494		
scpnrg3				✓
scpnrg4		1.7857		
scpnrg5				✓

**Table 15:** Comparative RPD performance across metaheuristics over 45 instances. Data taken from [36].

MH	Avg Min RPD	Best Instances	Avg Rank
BDOA	0.02	42	1.00
BAOA	1.47	21	2.41
SCA	2.28	15	3.02
PSA	1.80	6	3.45
GWO	2.12	2	4.01
BGO	2.15	1	4.11

**Table 16:** Shapiro–Wilk normality test results per SCP instance (31 independent runs).

Instance	N	W	*p* Value	Normal
41	31	0.7532	0.0000	No
410	31	0.4361	0.0000	No
42	31	0.6493	0.0000	No
43	31	0.6861	0.0000	No
44	31	0.5284	0.0000	No
45	31	0.7279	0.0000	No
46	31	0.8348	0.0002	No
47	31	0.3829	0.0000	No
48	31	0.4829	0.0000	No
49	31	0.6508	0.0000	No
51	31	0.5672	0.0000	No
510	31	0.3967	0.0000	No
52	31	0.8729	0.0016	No
53	31	0.7911	0.0000	No
54	31	0.6122	0.0000	No
55	31	0.6617	0.0000	No
56	31	1.0000	1.0000	Yes
57	31	0.7251	0.0000	No
58	31	0.4890	0.0000	No
59	31	0.4152	0.0000	No
61	31	0.6606	0.0000	No
62	31	0.7869	0.0000	No
63	31	0.5470	0.0000	No
64	31	1.0000	1.0000	Yes
65	31	0.6065	0.0000	No
a1	31	0.8979	0.0064	No
a2	31	0.7756	0.0000	No
a3	31	0.8569	0.0007	No
a4	31	0.8787	0.0022	No
a5	31	0.8000	0.0001	No
b1	31	0.3444	0.0000	No
b2	31	0.2698	0.0000	No
b3	31	0.6068	0.0000	No

**Table 17:** Shapiro–Wilk normality test results per SCP instance (31 independent runs).

Instance	N	W	*p* Value	Normal
b4	31	0.4577	0.0000	No
b5	31	1.0000	1.0000	Yes
c1	31	0.8863	0.0033	No
c2	31	0.8661	0.0011	No
c3	31	0.7939	0.0000	No
c4	31	0.9057	0.0100	No
c5	31	0.8311	0.0002	No
d1	62	0.7989	0.0000	No
d2	62	0.7205	0.0000	No
d3	62	0.8024	0.0000	No
d4	62	1.0000	1.0000	Yes
d5	62	0.1714	0.0000	No
nre1	31	1.0000	1.0000	Yes
nre2	31	0.7051	0.0000	No
nre3	31	0.5470	0.0000	No
nre4	31	0.7044	0.0000	No
nre5	29	1.0000	1.0000	Yes
nrf1	31	0.1756	0.0000	No
nrf2	31	1.0000	1.0000	Yes
nrf3	31	0.6377	0.0000	No
nrf4	31	0.4448	0.0000	No
nrf5	31	0.3974	0.0000	No
nrg1	31	0.9061	0.0103	No
nrg2	31	0.9320	0.0498	No
nrg3	31	0.9355	0.0621	Yes
nrg4	31	0.9060	0.0102	No
nrg5	8	0.8658	0.1370	Yes
nrg5	31	0.9110	0.0137	No
nrh1	31	0.9053	0.0098	No
nrh2	31	0.8220	0.0001	No
nrh3	31	0.2719	0.0000	No
nrh4	31	0.8790	0.0022	No
nrh5	28	0.5319	0.0000	No

**Table 18:** Wilcoxon signed-rank test results for DOA compared with other metaheuristics based on RPD values.

Comparison	Wins	Ties	Losses	*p*-Value
DOA vs. AOA	40	5	0	$3.56\times 10^{-8}$
DOA vs. SCA	38	7	0	$7.73\times 10^{-8}$
DOA vs. PSA	38	7	0	$7.73\times 10^{-8}$
DOA vs. GWO	38	7	0	$7.73\times 10^{-8}$
DOA vs. BGO	40	5	0	$3.57\times 10^{-8}$

**Table 19:** Average ranking and effect size (Cliff’s delta) of DOA compared to other metaheuristics.

Algorithm	Average Rank	Cliff’s Delta (vs. DOA)
DOA	**1.34**	–
AOA	4.30	0.873
SCA	3.76	0.829
PSA	3.68	0.831
GWO	3.97	0.831
BGO	3.96	0.879

## Data Availability

The original contributions presented in the study are included in the article; further inquiries can be directed to the corresponding authors.

## Abbreviations

The following abbreviations are used in this manuscript:

SCP	Set Covering Problem
DOA	Dream Optimization Algorithm
REM	Rapid Eye Movement
BDOA	Binary Dream Optimization Algorithm
AOS	Adaptive Operator Selection
BSRO	Bandit Selection of Repair Operator
AOA	Arithmetic Optimization Algorithm
DS	Discretization Scheme
RPD	Relative Percentage Deviation
CV	Coefficient of Variation
SCA	Sine Cosine Algorithm
PSA	Pendulum Search Algorithm
GWO	Grey Wolf Optimizer
BGO	Binary Growth Optimizer
ACO	Ant Colony Optimization
V3-ELIT	V-shaped elitist binarization
